# Effects of combining diaphragm training with electrical stimulation on pain, function, and balance in athletes with chronic low back pain: a randomized clinical trial

**DOI:** 10.1186/s13102-021-00250-y

**Published:** 2021-03-04

**Authors:** Khadijeh Otadi, Noureddin Nakhostin Ansari, Shahriar Sharify, Zahra Fakhari, Hadi Sarafraz, Amir Aria, Omid Rasouli

**Affiliations:** 1 Department of Physiotherapy, School of Rehabilitation, Tehran University of Medical Sciences, Tehran, Iran; 2 Occupational Medicine Department, Hormozgan University of Medical Sciences, Bandar Abbas, Iran; 3 Prana Physiotherapy Clinic, Tehran, Iran; 4 Department of Public Health and Nursing, Faculty of Medicine and Health Sciences, Norwegian University of Science and Technology, Trondheim, Norway

**Keywords:** Back pain, Diaphragm muscle, Breathing exercises, Transcutaneous electrical nerve stimulation

## Abstract

**Background:**

It is unknown how diaphragm training combined with electrical stimulation affects pain, function, static stability, and balance in athletes with chronic low back pain (CLBP). This study aimed to explore the effects of combining diaphragm training with electrical stimulation on pain, function, static stability, and dynamic balance in athletes with nonspecific CLBP.

**Methods:**

The design was a randomized clinical trial. A total of 24 amateur athletes (12 women, 12 men, mean age: 35.2 ± 9.8) with nonspecific CLBP were randomly allocated into two groups. The experimental group (*n* = 12) received diaphragm training plus Transcutaneous Electrical Nerve Stimulation (TENS), while the control group (*n* = 12) received TENS alone. Both groups underwent 12 sessions over a four-week period. Static stability, dynamic balance, pain, and function were measured pre- and post-intervention.

**Results:**

Analysis of variance 2 × 2 revealed greater improvements in pain (*p* < 0.001), static stability (*p* < 0.001), and dynamic balance (*p* < 0.01) in the experimental group compared to the control group. Function was improved in both groups following the interventions (*p* < 0.001), and there was a trend of a larger improvement in the experimental group than the control group (*p* = 0.09). Fisher’s exact test showed that the experimental group reported ≥50% improvement only in the pain score, not function, compared to the group that received TENS alone (*p* = 0.005).

**Conclusions:**

Pain, function, static stability, and dynamic balance were improved in both groups following 12 intervention sessions. However, pain, static stability, and dynamic balance were improved to a greater extent in diaphragm training plus TENS than TENS alone in amateur athletes with CLBP. Therefore, it seems beneficial to add diaphragm training to the rehabilitation program for athletes with nonspecific CLBP.

**Trial registration:**

The trial was retrospectively registered in the Iranian Registry of Clinical Trials (www.irct.ir) on September 10, 2020 as IRCT20090228001719N8.

## Background

Low Back Pain (LBP) in athletes is a common source of pain, and many athletes experience LBP [1, 2]. The transversus abdominis (TrA), internal oblique (IO), multifidus, diaphragm, and pelvic floor muscles are considered as deep trunk muscles, which provide stability and motor control to the spine [3, 4]. This spinal stability is crucial for correctly performing limb and trunk movements, particularly in athletes [4]. Previous research has shown reduced spine stabilization, lumbar segmental instability, and decreased control of the deep trunk muscles in athletes who suffered from LBP [1, 5, 6]. The diaphragm muscle is a respiratory muscle with postural function [7, 8]. Patients with chronic LBP (CLBP) are more susceptible to diaphragm fatigue than healthy people and, therefore, can likely benefit from exercises designed to improve strength/endurance in this muscle [9]. A recent study also reported reduced diaphragm thickness in athletes who suffered from LBP compared to healthy athletes [5]. Another study demonstrated that 8 weeks of diaphragm training resulted in increased diaphragm thickness and other stabilizer muscles in the lumbar region [10]. These novel findings indicate that diaphragm training may be an additional useful intervention for athletic performance, prevention of injury, and rehabilitation to improve respiratory capacity, torso stability, and balance. Collectively, these improvements might also reduce LBP occurrence [5, 11–14].

Balance is essential for performing everyday activities and enhances physical readiness for better sports performance. Poor scores on balance tests are directly linked to increased injury rates in a healthy athletic population [15]. Increasing spinal stability has been reported to reduce lower back injuries and improve static and dynamic balance in athletes [16]. Therefore, strengthening the deep trunk muscles is essential to improve spinal instability in athletic physical performance [17].

Conventional conservative treatment for CLBP focuses on electrotherapy, exercise therapy, and manual therapy [18]. Treatment of CLBP with Transcutaneous Electrical Nerve Stimulation (TENS) results in significant pain reduction [19]. Sayilir and Yildizgoren (2017) showed that using TENS for patients with CLBP could reduce pain and improved physical functions; hence, they suggested using TENS as part of the rehabilitation CLBP [20]. Also, spinal exercise therapy has been recommended to focus on muscle activation, neuromuscular control, static and dynamic stability [3]. Although data are still limited, recent studies reported that diaphragm training may be beneficial for spinal stability and posture [13, 21].

However, to our knowledge, it is unknown how diaphragm training combined with electrical stimulation affects pain, function, stability, and balance in athletes with CLBP. Therefore, the current study aimed to explore the effects of combining diaphragm training with TENS on pain, function, static stability, and dynamic balance in athletes with nonspecific CLBP. The main hypothesis was that combining diaphragmatic training with TENS would lead to greater improvements in pain level, function, static stability, and dynamic balance compared to using TENS alone in amateur athletes with nonspecific CLBP.

## Methods

### Design

This single-blinded, randomized clinical trial was conducted between July 2019 and January 2020. This study was conducted based on the Guidelines for Consolidated Standards of Reporting Trials (CONSORT).

### Participants

G*power 3 was used to calculate the sample size. A sample size of 24 participants (including 20% dropout) was capable of detecting a significant difference in the pain score between the groups according to a similar study [10], assuming a significance level of 0.05, power of 80%, and medium effect size (*d* = 0.5).

**Fig. 1:**
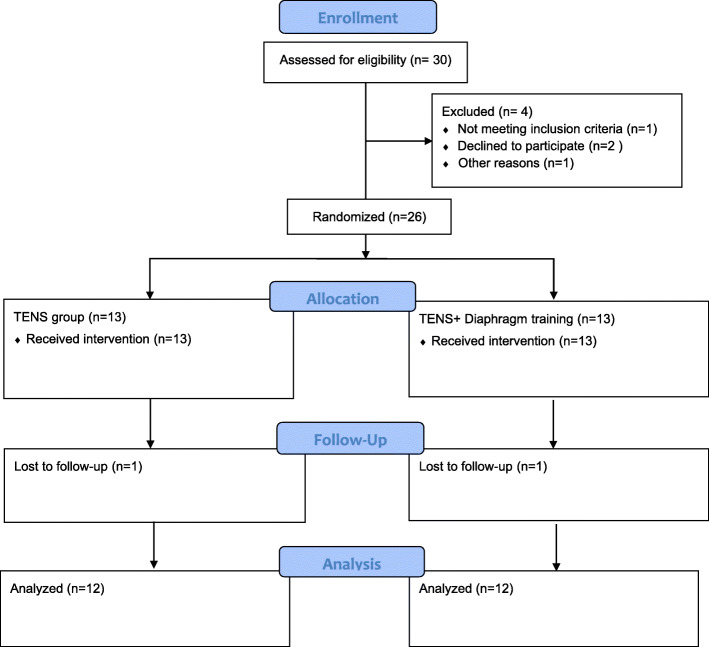
Flow diagram of included participants

A total of 24 amateur athletes aged 20–50 years were recruited from two outpatient rehabilitation clinics (Table 1). Participants were included if they had intermittent nonspecific CLBP for ≥12 weeks, with VAS between 3 to 7, [22]. All participants were active at a recreational level, for 2–4 times per week, since at least 3 years. Eighteen participants regularly engaged in strength training, and 6 participants did aerobic exercise. LBP is classified as nonspecific LBP when there is no known specific pathology [23]. Participants were excluded if they had lumbar surgery experience, inflammatory spinal disease, spinal deformities, or neurologic radiating pain. Participants were also excluded from the study if they were unable to perform exercises. Participants were randomly assigned to the following two groups by block randomization method: TENS treatment or TENS plus diaphragm training (Fig. 1). Both groups underwent 12 intervention sessions over 4 weeks (3 sessions weekly; odd or even days).

**Table 1:** Anthropometric characteristics of participants

Variable	Control (***n*** = 12)	Experimental (***n*** = 12)
**Gender**	7 females, 5 males	5 females, 7 males
**Age (year)**	34.2 ± 10.8	36.2 ± 8.9
**Height (m)**	1.7 ± 0.6	1.75 ± 0.1
**Weight (kg)**	65.1 ± 25.7	66.2 ± 27.8
**BMI (kg/m**^**2**^**)**	24.9 ± 3.1	25.8 ± 5.7

All participants received information about the study procedure, and they gave written informed consent before entering into the study. The study protocol was approved by the Research Ethics Committee at the Tehran University of Medical Sciences (IR.TUMS.FNM.REC.1398.138) and followed the principles of the Declaration of Helsinki. The trial was retrospectively registered in the Iranian Registry of Clinical Trials (www.irct.ir) on September 10, 2020 as IRCT20090228001719N8.

### Outcome measurements

The following outcomes were collected by the examiner or self-report questionnaires.

#### Pain

Pain level was assessed by the Numerical Rating Scale (NRS). The participants rated their pain on a defined 0–10 scale, where 0 is no pain, and 10 is the worst pain imaginable. NRS has different advantages, such as simplicity, reproducibility, and sensitivity to small changes in pain [24]. NRS takes less than 1 min to complete and is a valid and reliable scale to measure pain intensity [25].

#### Function

Core Outcome Measures Index (COMI) is a short, self-reported questionnaire for assessing important outcomes in patients with LBP [26]. The COMI covers five different domains, with seven individual items: pain intensity (two separate items measuring back pain and leg/buttock pain), back function in everyday life (one item), symptom-specific well-being (one item), the general quality of life (one item) and disability (two separate items measuring social disability and work disability). The composite COMI score (range 0–10) is calculated using the average score of the five domains, and higher COMI scores indicate worse status [26]. For the domain pain intensity, the data are collected using 0–10 graphic rating scales, with the higher of the two values for back pain and leg/buttock pain being used to represent the “pain” domain. Five-point scales (1–5) are used for the remaining domains, with the scores being rescaled into a 0–10-point range (score (1–5) minus 1, multiplied by 2.5). The values for the two disability items are averaged to represent the “disability” domain. Previous studies documented its reliability, validity, sensitivity to change [26, 27]. Reliability and validity of the Persian version of COMI was also reported by Ansari et al. [28].

#### Static stability

**Fig. 2:**
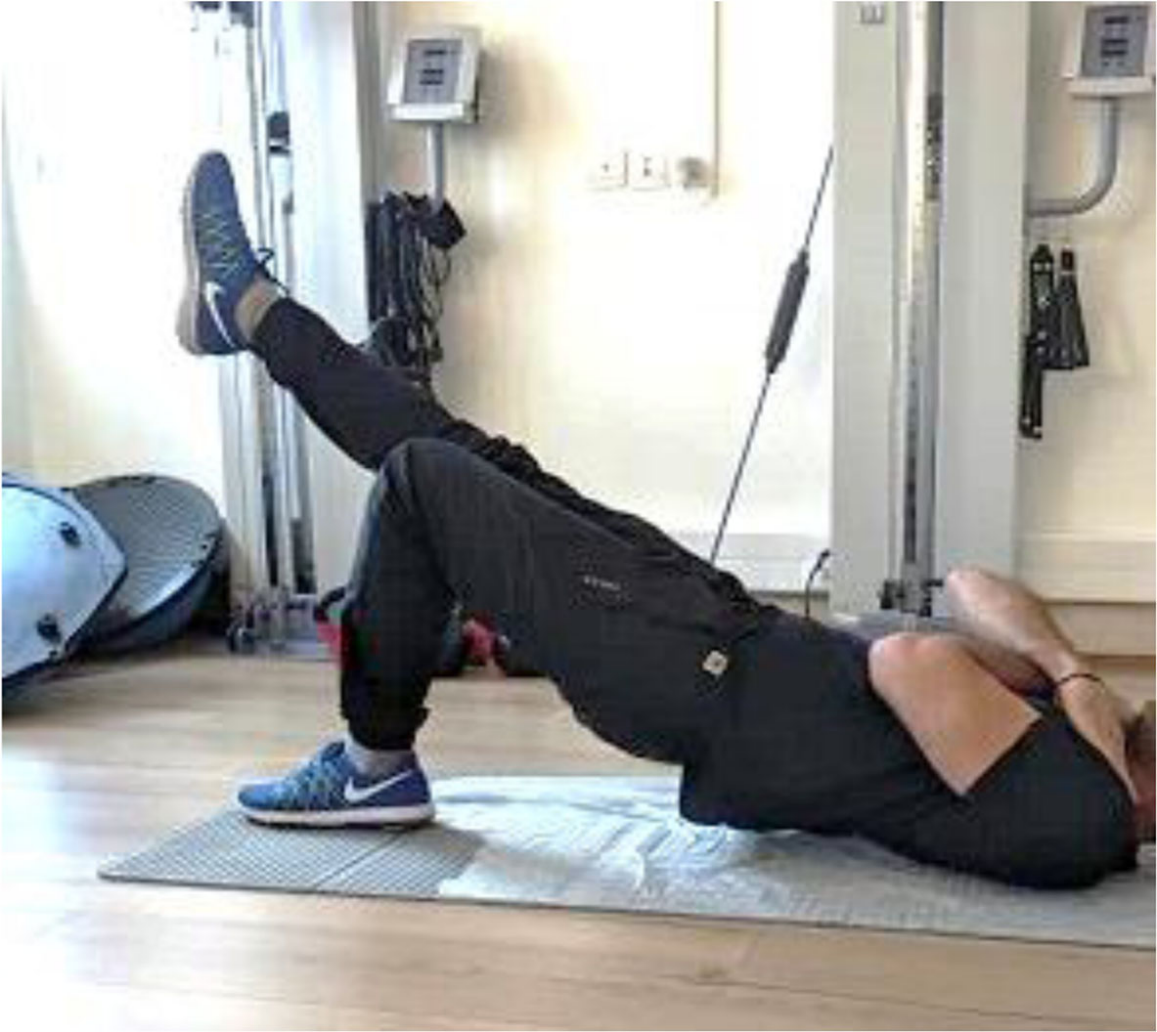
Unilateral Hip Bridge Endurance test (UHBE)

Static stability was determined using the Unilateral Hip Bridge Endurance test (UHBE). UHBE test is considered a simple is a clinical measure of spinal stability [29]. It was performed with the participants lying supine with their arms across their chest, knees in flexion, and feet flat on the table. The participants performed a double-leg hip bridge, and once a neutral spine and pelvis position was achieved, the participants were instructed to extend one knee (randomly determined) so their leg was straight, and their thighs were parallel to one another. Participants were instructed to hold this position as long as possible. The test was terminated when they were no longer able to maintain a neutral pelvic position, as noted by a 10-degree change in transverse or sagittal plane alignment. Pelvic positioning in the transverse plane was monitored by a digital inclinometer attached to a mobilization belt that was tightly secured to the individual’s pelvic (Fig. 2). The validation of this test was previously reported [29].

#### Dynamic balance

Star Excursion Balance Test (SEBT) was performed to assess dynamic balance [30]. The SEBT has been reported as a reliable, valid test for assessing dynamic postural-control deficits and outcomes in lower extremity injury [31]. Following previous studies, the participants were instructed to stand on the dominant leg and, with the tip of the great toe of the non-stance limb extending along a line as far as possible towards the anterior, posteromedial, and posterolateral directions [30, 32]. Participants held their hands on the iliac crest during testing. The order of directions was randomized, and for each direction, participants performed six practices followed by a 2-min rest and three test trials afterward. The average of three reaching distances was calculated and used for analysis. The test was rejected, and participants had to repeat it if they failed to return the reaching limb to the starting point or failed to maintain a unilateral stance, lifted, or moved the standing foot from the line. To standardized test results, reaching distance was normalized by the participant’s leg length. The leg length was measured from the distal end of the anterior superior iliac spine to the lateral malleolus’s distal end on that limb [33]. Excellent reliability has been reported for this test [34].

### Intervention

**Fig. 3:**
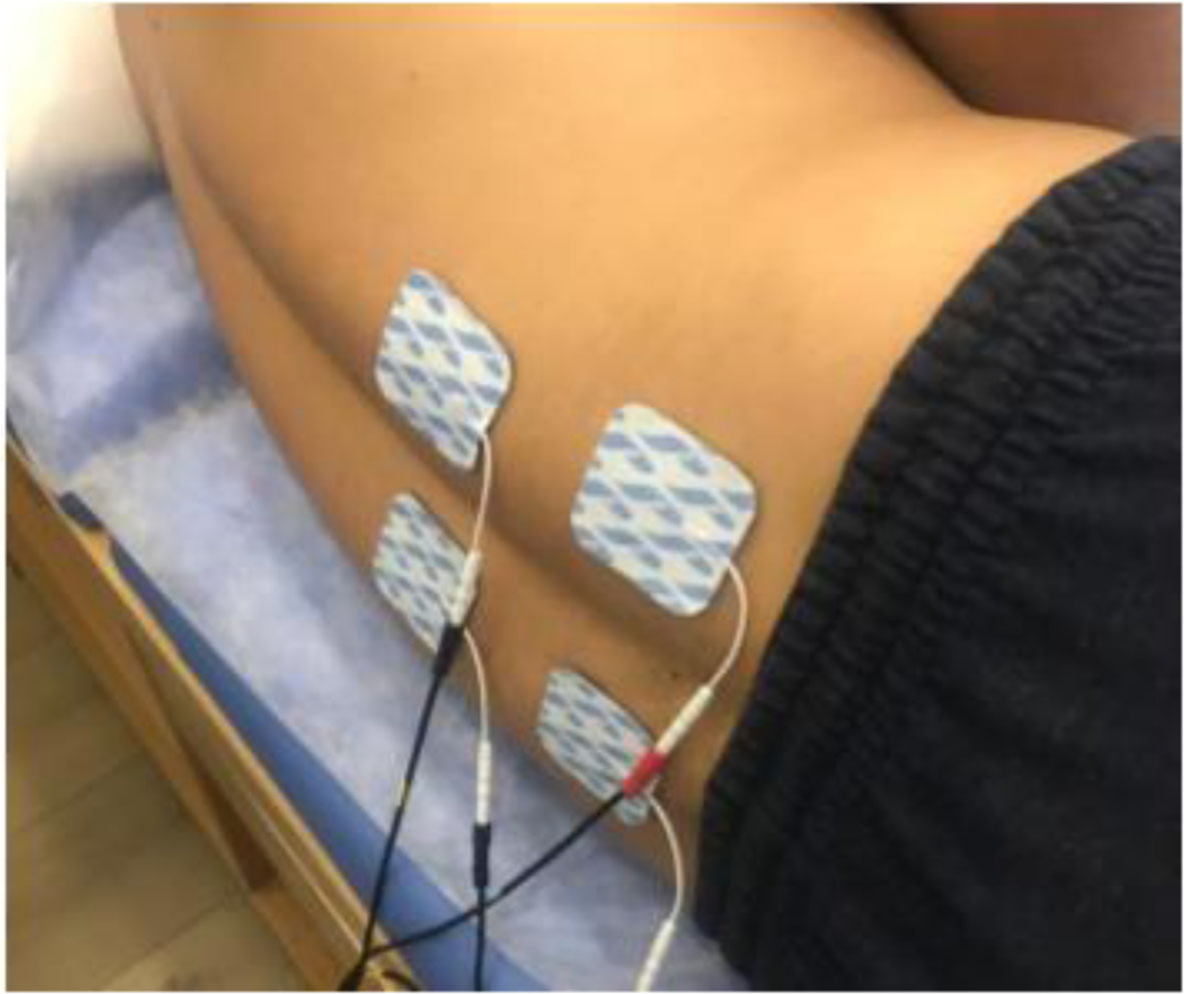
The participants received TENS in a side-lying position with flexed hips and knees

All 24 participants completed the NRS, COMI, UHBT, and SEBT at baseline (2–3 days before the first intervention session) and after completing the 4-week intervention (2–3 days after the last (12th) intervention session. The control group received three sessions (30 min conventional TENS, impulse duration: 100 μs, frequency: 100 HZ) per week for 4 weeks [35]. The experimental group received TENS (the same setting as the controls) plus diaphragm training for 12 sessions (on odd or even days) over a 4-week period. The participants in both groups received TENS in a side-lying position with flexed hips and knees (Fig. 3). All participants in both groups also received similar patient education information during the sessions. A physiotherapist supervised diaphragmatic exercises at the beginning of each week to ensure that each exercise was performed correctly. Participants were assigned two exercises per week and asked to complete each exercise for 5 min, twice daily, for a total of 20 min per day at least 5 days per week. Instruction and feedback were given to participants on assessment days. When participants attended a clinic, diaphragmatic exercises were performed after electrotherapy with the physiotherapist.

**Fig. 4:**
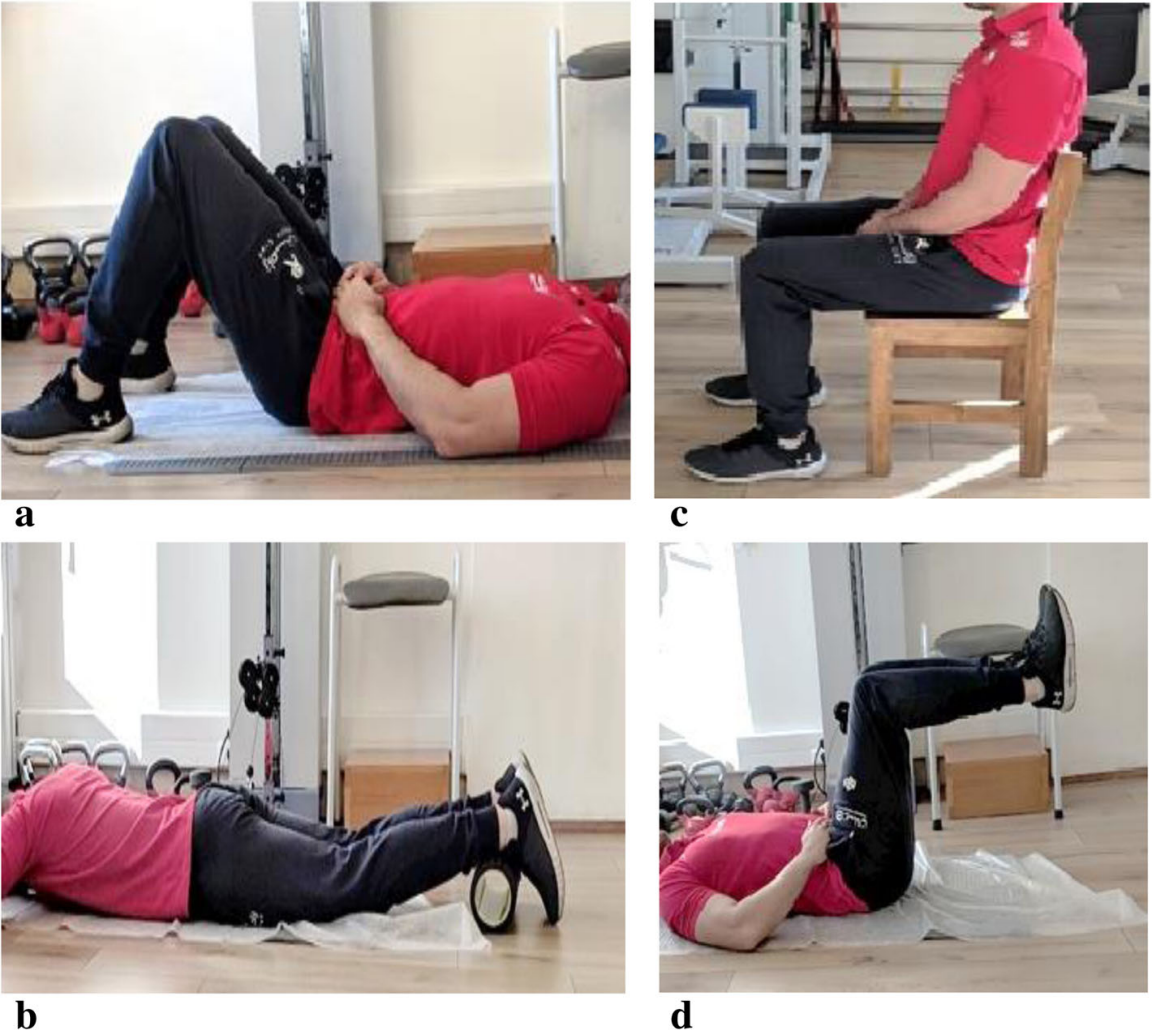
**a** Supine Breathing. **b** Crocodile breathing. **c** Seated breathing. **d** 90/90/90 breathing

The exercises planned for each week were as follows:

- ***First week:*** Supine breathing + Crocodile breathing
- ***Second week:*** Supine breathing with TheraBand + Crocodile breathing with TheraBand
- ***Third week:*** Seated breathing + 90/90/90 breathing
- ***Fourth week*:** Seated breathing with TheraBand + 90/90/90 breathing with TheraBand
- ***Supine Breathing:*** Participants were instructed to lay supine in a hook-lying position and arms in a comfortable position. They were asked to focus on breathing with their diaphragm, the breath filling into their lower abdomen and posterior chest wall. They were asked to keep their ribs depressed and keep their shoulders and neck relaxed. During resisted exercise, TheraBand was added around the thoracolumbar junction and fixed by the therapist to a distal section of the bed to enhance resistance (Fig. 4a) [21].
- ***Crocodile breathing:*** Participants were instructed to lay prone with their hands in a diamond shape supporting their forehead. They were asked to push their ribs out laterally and breathe all the way down to the sacrum. A TheraBand was placed under the participant’s thoracolumbar junction during resistance training (Fig. 4b) [21].
- ***Seated breathing:*** Participants were seated on a hard surface with their knees, hip, and ankles, all at 90°. They were told to sit tall as if a “string was pulling them up from the top of their head” while maintaining all previously discussed breathing cues: preventing lower rib flair, breathing deeply, and relaxing their shoulders, neck, and arms (Fig. 4c) [21].
- ***90/90/90 breathing:*** Participants were placed in the 90/90/90 position and were asked to hold their legs while maintaining all previously discussed breathing cues: controlling their ribs and thoracolumbar junction, breathing deeply, relaxing their shoulders, neck, and arms (Fig. 4d) [21]. During resistance training, a TheraBand or belt was placed under the participant’s thoracolumbar junction. The participant was instructed to prevent the examiner from pulling the belt away. At home, participants were instructed to tie the TheraBand around a table or chair and leave tension in it to simulate the effect of pulling.

### Statistical analysis

Data were analyzed using SPSS version 22.0 (IBM, USA). The normal distribution of data was confirmed by the Shapiro-Wilk test and residual plot assessment. Descriptive results were presented as mean ± standard deviation. ANOVA 2 × 2 (time x group) was used to compare the continuous outcomes in groups before and after the intervention, with the group as the between-subjects factor (*n* = 2, TENS and TENS+DT) and time (*n* = 2; pre-intervention and post-intervention) as the within-subjects factor. Partial eta-squared (*η2p*) was used for effect size. We used Fisher’s exact test to compare the proportion of participants with a greater than 50% improvement in pain and function. The statistical significance level was set as α = 0.05.

## Results

The anthropometric characteristics of the participants in each group are shown in Table 1. There were no significant differences between the groups at baseline. Table 2 lists outcome values at baseline and post-intervention for each group.

**Table 2:** Mean ± standard division for outcomes at baseline and post-intervention for each group

Variable		Group		Pre-intervention	Post-intervention
**Pain (NRS)**		**TENS**		4.7 ± 1.3	3.1 ± 1.3
		**TENS + DT**		5.5 ± 1.1	1.7 ± 0.6
**Function (COMI)**		**TENS**		4.5 ± 0.9	2.6 ± 0.6
		**TENS + DT**		4.5 ± 0.7	1.9 ± 0.9
**Static stability (UHBE)**		**TENS**	**Rt**	25.9 ± 11.5	27.3 ± 11.5
			**Lt**	25.1 ± 11.1	28.3 ± 13.7
		**TENS + DT**	**Rt**	26.3 ± 11.1	37.5 ± 12.6
			**Lt**	25 ± 10.1	40.1 ± 10.6
**Dynamic Balance (SEBT)**	**Ant**	**TENS**	**Rt**	81.8 ± 9.8	82.35 ± 10.7
			**Lt**	82.2 ± 10.6	83.8 ± 10.7
		**TENS + DT**	**Rt**	80.1 ± 7.1	87.7 ± 7.9
			**Lt**	81.7 ± 7.8	86.3 ± 8.3
	**Post Lat**	**TENS**	**Rt**	76.4 ± 13.1	82.1 ± 14.6
			**Lt**	76.9 ± 14.5	78.7 ± 14.1
		**TENS + DT**	**Rt**	71.5 ± 7.68	91.5 ± 8.3
			**Lt**	69.7 ± 7.1	76.9 ± 8.6
	**Post Med**	**TENS**	**Rt**	83.2 ± 14.9	85.5 ± 14.3
			**Lt**	83.4 ± 13.8	88.7 ± 17.8
		**TENS + DT**	**Rt**	79.5 ± 7.3	98.1 ± 8.7
			**Lt**	77.1 ± 8.4	83.9 ± 10.1

*TENS* Trans Cutaneous Electrical Nerve Stimulation, *DT* Diaphragm training, *NRS* Numeric Rating Scale, *COMI* Core Outcome Measure Index, *UHBE* Unilateral Hip Bridge Endurance test, *SEBT* Star Excursion Balance Test, *Ant* Anterior, *PostLat* Posterior lateral, *PostMed* Posterior medial, *Rt* Right, *Lt* Left

### Pain and function measures

As shown in Table 3, the ANOVA 2 × 2 showed a significant interaction between time × group (η2*p* = 0.52, *p* < 0.001) for pain (NRS), indicating a greater decrease in pain score in the experimental group.

**Table 3:** The results of ANOVA 2 × 2 for the variables

Variable		Main effect group		Main effect time	Interaction group × time
**Pain (NRS)**		F(1, 22) = 0.4, *p* = 0.5		F(1, 22) =119.3, *p* < 0.001	F(1, 22) =19.8, *p* < 0.001
**Function (COMI)**		F(1, 22) = 1.1, *p* **=** 0.3		F(1, 22) =109.5, *p* < 0.001	F(1, 22) = 2.4, *p* = 0.09
**Static stability (UHBE)**		**Rt**	F(1, 22) =1.3, *p* = 0.3	F(1, 22) =3.8, *p* = 0.06	F(1, 22) = 6.3, *p* = 0.02
		**Lt**	F(1, 22) =1.3, *p* = 0.2	F(1, 22) = 67.8, *p* < 0.001	F(1, 22) = 28.2, *p* < 0.001
**Dynamic Balance (SEBT)**	**Ant**	**Rt**	F(1, 22) =0.9, *p* = 0.3	F(1, 22) =128.7, *p* < 0.001	F(1, 22) = 39.6, *p* < 0.001
		**Lt**	F (1, 22) = 0.2, *p* = 0.6	F (1, 22) = 128.6, *p* < 0.001	F (1, 22) = 7.9, *p* = 0.01
	**Post Med**	**Rt**	F(1, 22) =1.0 *p* = 0.3	F(1, 22) =36.7, *p* < 0.001	F(1, 22) = 22.8, *p* < 0.001
		**Lt**	F (1, 22) = 0.9, *p* = 0.3	F (1, 22) = 21.5, *p* < 0.001	F(1, 22) = 0.4, *p* = 0.5
	**Post Lat**	**Rt**	F(1, 22) =0.2, *p* = 0.6	F(1, 22) =88.5, *p* < 0.001	F(1, 22) = 27.9, *p* < 0.001
		**Lt**	F (1, 22) = 0.8, *p* = 0.4	F(1, 22) = 8.8, *p* = 0.008	F(1, 22) = 3.25, *p* = 0.08

*TENS* Trans Cutaneous Electrical Nerve Stimulation, *DT* Diaphragm training, *NRS* Numeric Rating Scale, *COMI* Core Outcome Measure Index, *UHBE* Unilateral Hip Bridge Endurance test, *SEBT* Star Excursion Balance Test, *Ant* Anterior, *PostLat* Posterior lateral, *PostMed* Posterior medial, *Rt* Right, *Lt* Left

Regarding function (COMI), both groups experienced an improvement in function, and there was a significant main effect for time (η2p = 0.9, *p* < 0.001). Whilst the experimental group was observed to have a slightly larger improvement over time (Table 2), there was not a statistically significant interaction between group and time (*p* = 0.09) (Table 3).

Considering ≥50% improvements in the pain and function scores, Fisher’s exact test showed that the experimental group reported ≥50% improvement only in the pain score, not function, than TENS alone (*p* = 0.005).

### Static stability and dynamic balance

We observed improvements in static stability and dynamic balance after completing the four-week intervention in both groups (Table 3). There were significant interactions of time × group for static stability (UHBE) on the left side (η2*p* = 0.6, *p* < 0.001) and the right side (η2*p* = 0.3, *p* = 0.02), demonstrating significantly greater improvements on both sides in the experimental group.

Regarding dynamic balance (SEBT), ANOVA 2 × 2 showed interaction of time ×group on the right side in the anterior direction (η2p = 0.7, *p* < 0.001), posteromedial direction (η2p = 0.55, *p* < 0.001) and posterolateral direction (η2p = 0.61, *p* < 0.001). A significant interaction of time ×group for dynamic balance (SEBT) was also found only on the left side in the anterior direction (η2p = 0.3, *p* = 0.01). These findings indicate greater improvements for dynamic balance in the experimental group compared to the control group (Table 3).

## Discussion

This study investigated whether combing diaphragm training with electrical stimulation improves pain, function, static stability, and dynamic balance in amateur athletes with nonspecific CLBP. Our main finding was that when diaphragm training was added to TENS during a 12-session intervention, the pain was reduced, static stability and dynamic balance were improved to a greater extent compared to TENS alone. We also observed a trend of a larger improvement in the function score following a combination of diaphragm training and TENS; however, this result was not statistically significant. Thus, the findings partially support our main hypothesis that combining diaphragmatic training with TENS would lead to greater improvements in pain level, static stability, and dynamic balance compared to using TENS alone in athletes with nonspecific CLBP.

### Pain

Both groups showed significantly reduced pain after undergoing 12 intervention sessions compared to the pre-intervention scores. However, the reduction of pain in the experimental group was greater than in the control group. A 30% change in pain score from baseline is clinically significant for individual patients with LBP [36]. The reduction was approximately 34 and 69% in the control and experimental group, respectively. This degree of pain reduction that we observed is in line with previous findings [37]. The positive effect of TENS on the reduction of pain in participants with CLBP was concluded by one systematic review [37]. Likewise, other researchers found that sensory electrical stimulation had a greater effect than other currents on reducing pain [35]. The reduction in pain can be due to the local effect of electrical currents on the nociceptor of the lumbar region as well as the reduction of back muscle spasms [35, 37]. However, being that we observed a greater reduction of pain in the experimental group, it appears that diaphragm training augments what can be achieved with TENS alone. The diaphragm is the primary muscle of inspiration but also is involved in trunk stability and control of posture as part of the deep trunk muscles [13]. Accordingly, previous studies have shown that breathing training can reduce pain in LBP due to increased stability of the trunk as well as reduced tension in other central muscles of the body, especially the multifidus muscle [9, 11]. Janssens et al. (2015) reported that 8 weeks of high-intensity inspiratory muscle training, albeit not low-intensity, effectively reduce pain and increase respiratory function in patients with CLBP [12]. Deep breathing exercise can also lead to improvements in deep trunk muscle activation and respiratory function during breathing in individuals with CLBP [38, 39]. Therefore, adding breathing exercise to electrotherapy in rehabilitation programs leads to more pain relief in athletes with CLBP.

### Function

The quality and function of daily activities measured by the COMI revealed significant improvements after completing 12 intervention sessions in both groups, but no significant difference was found in improvement observed between the two groups. COMI is a reliable, valid, and brief instrument to assess pain intensity, function, symptom-specific well-being, disability, and general quality of life in patients with back problems [28, 40]. Some questions of the COMI questionnaire are related to the quality of life, performance, and satisfaction of patients with LBP; therefore, psychological situations may affect the COMI score [41]. In this study, pain score was reduced by 69 and 34% in the experimental and control group, respectively. Therefore, they could return to their daily activities, and they answered the questions in COMI positively. In line with our results, previous studies have reported the positive effect of electrotherapy (e.g., TENS) on reducing disability in patients with CLBP measured by the Roland Morris questionnaire [35, 42]. Therefore, a reduction in pain can affect the patient’s psychological and physical aspects and may improve the quality of life of participants in both groups. As mentioned before, diaphragm training may increase diaphragm muscle strength and respiratory capacity and improve the spine’s stability. Consequently, performing diaphragm training can improve daily function in healthy individuals and patients with lumbar instability [21, 39, 43, 44]. For example, Mehling et al. (2005) reported significant improvements in function and pain with 6–8 weeks of breathing exercise compared to conventional physical therapy in patients with CLBP [11]. Our observation that the greater improvement in function that was present with the addition of diaphragm training to TENS did not reach statistical significance (*p* = 0.09) might be due to insufficient sample size.

### Stability and balance

After a 4-week intervention, static stability assessed by the UHBE was improved to a greater extent for both sides in the experimental group compared to the control group. This finding indicates that diaphragm training can lead to improved static stability. In agreement with our results, Stephens et al. (2017) also found improvement in static balance after an eight-week training intervention comprising diaphragmatic breathing in healthy individuals.

The diaphragm is considered as one of the deep trunk muscles that stabilize the trunk and spine during activities of upper and lower limbs, especially sports activities [17]. Increasing the stability of the trunk may consequently improve a person’s balance. Importantly, regarding the dynamic balance assessed by the SEBT, greater improvements were found in the experimental group (especially for the right side) compared to the control group. This finding could result from the effects of diaphragm training because it may positively affect intra-abdominal pressure, abdominal endurance, and movement efficiency, which, in turn, might have improved dynamic balance performance in the experimental group [45]. In addition, diaphragm training may enhance the activity of other deep trunk muscles; for example, Cho (2019) showed crocodile breathing is a good method to improve the multifidus muscle activity in patients with LBP [46]. Improving the trunk’s stability and more stability in the waist and pelvis can lead to improved balance in the lower limbs and even upper limb movements.

### Limitations

Some limitations should be considered while interpreting the findings of this study. A lack of a group with only diaphragm training was a major limitation. In addition, the sample of amateur athletes with CLBP limits the generalizability of the results to other groups and non-athlete patients. Another limitation was the possibility of unblinded examiner bias. A lack of follow-up assessment was another limitation. Moreover, we did not examine the test-retest reliability of the tests used in this study. Therefore, future studies are needed to investigate diaphragm training effects alone and with follow-up in different sample populations.

## Conclusions

In conclusion, this study suggests that diaphragm training plus TENS for 12 sessions can improve pain level, static stability, and dynamic balance to a greater extent than TENS alone in amateur athletes with nonspecific CLBP. There was also a trend of a larger improvement in function following a combination of diaphragm training and TENS, albeit not statistically significant. Therefore, it seems beneficial to add diaphragm training to the rehabilitation program for athletes with nonspecific CLBP.

## Data Availability

The datasets generated and analyzed during the current study are available from the corresponding author upon request.

## Abbreviations

CLBP: Chronic Low Back Pain
COMI: Core Outcome Measures Index
LBP: Low Back Pain
NRS: Numerical Rating Scale
SEBT: Star Excursion Balance Test
TENS: Transcutaneous Electrical Nerve Stimulation
UHBE: Unilateral Hip Bridge Endurance test
